# Accurate automatic object 4D tracking in digital in-line holographic microscopy based on computationally rendered dark fields

**DOI:** 10.1038/s41598-022-17176-1

**Published:** 2022-07-28

**Authors:** Mikołaj Rogalski, Jose Angel Picazo-Bueno, Julianna Winnik, Piotr Zdańkowski, Vicente Micó, Maciej Trusiak

**Affiliations:** 1 Warsaw University of Technology, Institute of Micromechanics and Photonics, 8 Sw. A. Boboli St., 02-525 Warsaw, Poland; 2 Departamento de Óptica y de Optometría y Ciencias de la Visión, Universitat de Valencia, C/Doctor Moliner 50, 46100 Burjassot, Spain

**Keywords:** Applied optics, Interference microscopy, Biomedical engineering, Optical metrology, Optical techniques, Imaging and sensing, Microscopy

## Abstract

Building on Gabor seminal principle, digital in-line holographic microscopy provides efficient means for space–time investigations of large volumes of interest. Thus, it has a pivotal impact on particle tracking that is crucial in advancing various branches of science and technology, e.g., microfluidics and biophysical processes examination (cell motility, migration, interplay etc.). Well-established algorithms often rely on heavily regularized inverse problem modelling and encounter limitations in terms of tracking accuracy, hologram signal-to-noise ratio, accessible object volume, particle concentration and computational burden. This work demonstrates the DarkTrack algorithm—a new approach to versatile, fast, precise, and robust 4D holographic tracking based on deterministic computationally rendered high-contrast dark fields. Its unique capabilities are quantitatively corroborated employing a novel numerical engine for simulating Gabor holographic recording of time-variant volumes filled with predefined dynamic particles. Our solution accounts for multiple scattering and thus it is poised to secure an important gap in holographic particle tracking technology and allow for ground-truth-driven benchmarking and quantitative assessment of tracking algorithms. Proof-of-concept experimental evaluation of DarkTrack is presented via analyzing live spermatozoa. Software supporting both novel numerical holographic engine and DarkTrack algorithm is made *open access*, which opens new possibilities and sets the stage for democratization of robust holographic 4D particle examination.

## Introduction

Digital holographic microscopy provides an efficient access to 3D object information through numerical refocusing an optical field^1^. Both amplitude and quantitative phase distributions can be noninvasively yielded^2,3^ to enhance the high-contrast label-free examination with sub-micrometer resolution^4^. Holographic solutions can thus serve as capable tools for particle tracking (PT)^5^ that is crucial in, e.g., analyzing cell biophysical processes^6^, flow measurements^7^, optofluidics^8^, microfluidics^9^, point-of-care diagnostics^10^ and optical manipulation^11^, as “particle” can mean any object from nano to micro scale, both biological and synthetic. In-line architectures based on Gabor principle^12^, although somewhat less popular than off-axis methods^5^, are especially interesting due to large space–time-bandwidth product and simple setups^13,14^. Lensless digital in-line holographic microscopy (DIHM)^15^ further advances these capabilities as it allows for compact setups to provide accurate analysis of very large volumes (even exceeding 100 mm^3^) without the limitations imposed by conventional imaging optics (field-of-view, depth of field, resolution, and aberrations). Described favorable features granted its success in PT^16^ based advances in biomedicine^14,17^ (e.g., air pollution examination^18^ and sperm analysis^19^), and technical sciences (e.g., colloidal particle velocimetry^7,16,20^).

Time–space tracking of multiple objects registered within a sequence of in-line holograms essentially comes down to determination of (x, y, z) coordinates for segmented particles and their temporal “linking”^6,7,21,22^. The (x,y) coordinates are often calculated using binarization and segmentation routines^5,19,22,23^, which are highly dependent on the object signal quality. The z coordinate is defined as the location of the plane of focus for a given object and is searched for via autofocusing algorithms^5,24–27^. This contribution is based on the DarkFocus “sharpness” metric^25^, as it allows for robust analysis of mixed phase-amplitude objects—we will use it in a novel localized way, however, alongside with new take on (x,y) position detection. It is worthy to note that machine learning based solutions for autofocusing^28,29^, 3D particle imaging^30,31^ and general data reconstruction^32^ revolutionized recently also the field of digital in-line holography. Those methods, although very capable and already well-established, need crucial training and are generally task-specific for a given dataset^28–32^ or require an initial guess or object/physics priors^33^. Important class of accurate and robust PT algorithms utilizes inverse problem solvers employing strong regularization and iterative optimization procedures^5,7,20,30,34–37^. Versatility of those approaches is governed by the extent of the model (e.g., Lorenz–Mie^21^) and the imposed priors. Additionally, the processing time is often greatly increased due to exhausting iterative optimization (computational load increases drastically with the concentration of particles and the investigated space^34–37^). Accurate (root-mean-square RMS error below the particle diameter^23,35^), fast, deterministic, model-free, versatile 4D tracking of dense (concentration > 10,000 particle/mm^3^^35^) volumes under realistic signal-to-noise ratio (SNR) can be treated as an important gap within the state-of-the-art of holographic PT. In this contribution, we propose DarkTrack—an *open-access* DIHM PT algorithm based on computationally generated high-contrast 3D dark fields and specialized data processing. An extra stand-out feature of DarkTrack is that it generates extended depth of focus (EDOF) 2D sequence of dynamic objects (without any learning process^28–32^).

In^19^ authors used similar image processing strategies based on binarization and segmentation, but with registering two holograms corresponding to different illumination angle, while the DarkTrack works with a single hologram. Moreover, dark field numerical reconstruction (propagation) was not considered in^19^. In^23^ binarization was performed on each slice from the reconstructed stack—in the DarkTrack we robustly and innovatively combine all slices of dark field stack to a single SNR-enhanced 2D image ready for binarization. Additionally, there is no background removal which limits the^23^ algorithm usage to the opaque samples. In^22^ background free holograms are used (background removal is based on computing of the difference between two consecutive holograms), however, no binarization nor segmentation nor object time-linking is used. The final reconstruction is given as a single 3D distribution—set of all detected 4D locations without the segmentation into objects and trajectories. Other reported DIHM 4D tracking approaches are based on deconvolution^20^, strong regularization^7,34^, neural networks^30,31^ or fitting the hologram to the model^21^, thus rely on priors which is not the case in model/learning-free DarkTrack.

Quantitative evaluation of tracking accuracy forms a very important challenge. It has been comprehensively addressed in fluorescence microscopy^38^, however the holographic microscopy field remains unsatisfied. Reported solutions are often based on thin object approximation and do not consider multiple scattering^39^, which makes them generally not fully feasible. Additionally, there is an accessibility problem as optics and photonics community lacks open access software for the efficient numerical simulation of a sequence of in-line holograms corresponding to a time-variant set of micro-objects exhibiting different trajectories. Tracking itself is insufficiently and only preliminarily democratized via a few available open-source codes—we discuss them in Supplement A. It is also worth mentioning that available solutions are based on particle steady flow priors, whereas the DarkTrack algorithm determines the trajectory regardless its complicated and abruptly changing path (which we will corroborate using randomized simulated trajectories and real life complicated 4D routs chosen by live spermatozoa). To address this important multidisciplinary challenge, we undertake a task of devising, implementing, and releasing to the community novel a numerical engine, i.e., MSHoloSim, which enables generation of a series of Gabor holograms of dynamic transient micro-objects occupying a predefined volume, while adhering to multiple scattering. We then use it to evaluate the proposed DarkTrack algorithm corroborating its accuracy under altered simulated conditions: hologram SNR and particle concentration. The MSHoloSim, openly available^40^, allows for setting particle size and trajectories, setup geometry and camera parameters.

## Description of proposed algorithmic solutions

**Figure 1:**
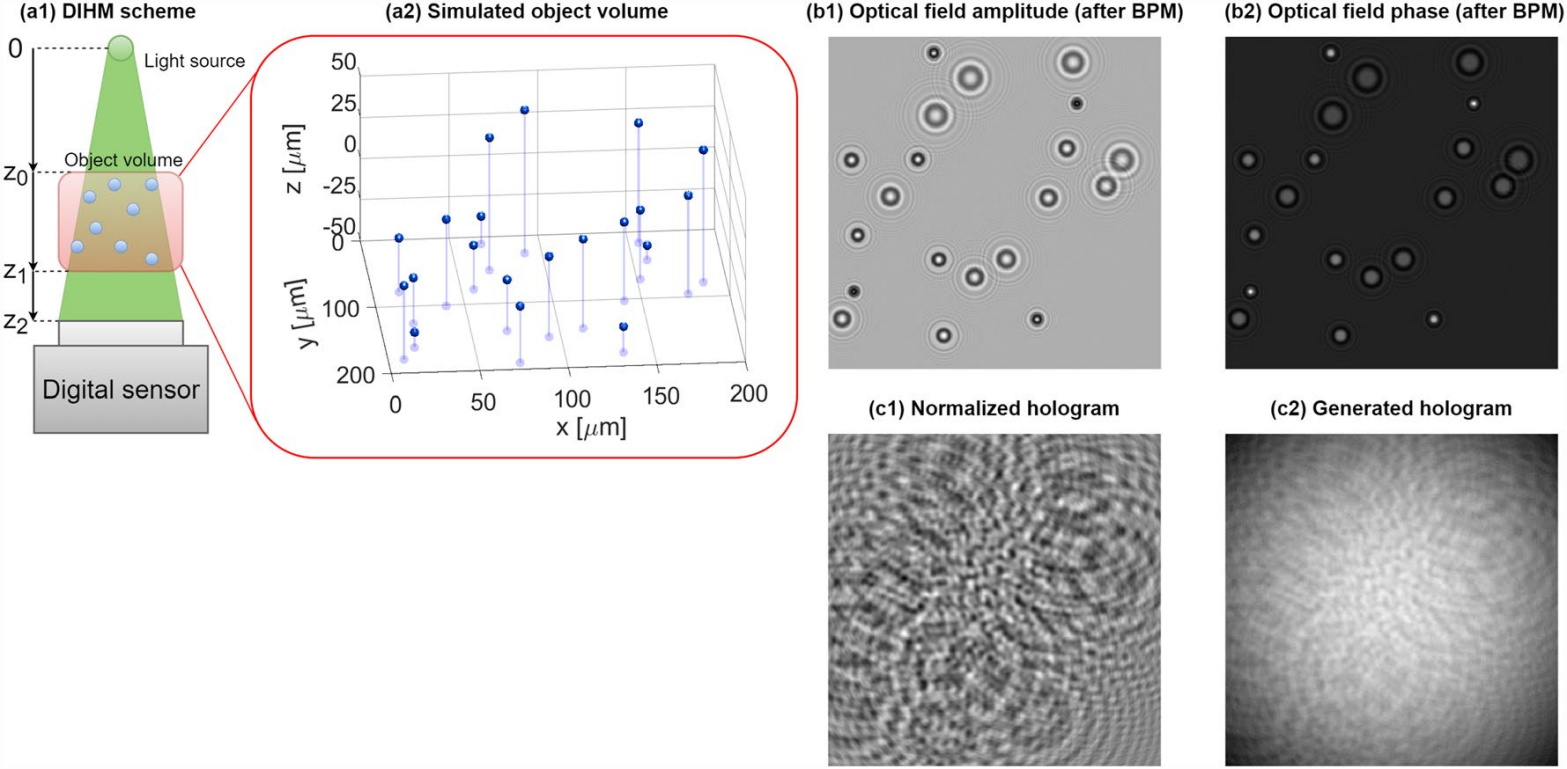
MSHoloSim engine working principle.

We first introduce the MSHoloSim, Fig. 1, as it constitutes the basis for devising and evaluating the DarkTrack algorithm. Our engine simulates a simple DIHM system Fig. 1a1. Process of generating a single hologram begins with simulating an object volume as a group of microbeads with user-defined complex refractive indices, diameters and *(x,* *y,* *z)* locations, Fig. 1a2. Then, we approximate that light reaches the object volume as a plane wave *u*_*z0*_*(x,* *y)* and use the state-of-the-art beam propagation method^41^ (see Supplement B) to propagate this optical field through the sample volume while accounting for multiple scattering, generating *u*_*z0*+*z1*_*(x,* *y)*, Fig. 1b. Next, we use the angular spectrum method (AS—Supplement C)^42^ to efficiently propagate *u*_*z0*+*z1*_*(x,* *y)* in the free space up to the detector plane, yielding *u*_*z*0+*z*1+*z*2_*(x,* *y)*. Finally, we generate the hologram H(x,y), Fig. 1c2, as:

1 $$H\left( {x,y} \right) = B\left( {x,y} \right) \times \left( {1 + \left| {u_{z0 + z1 + z2} \left( {x,y} \right)} \right|^{2} } \right) + N\left( {x,y} \right),$$

where $\left| {u_{z0 + z1 + z2} \left( {x,y} \right)} \right|^{2}$ is a normalized hologram, Fig. 1c1, $B\left( {x,y} \right)$ is the Gaussian background intensity component and $N\left( {x,y} \right)$ is a noise component. Supplying a set of temporally varying 3D coordinates, one receives a sequence of Gabor holograms closely following the physics of light propagation, scattering and imaging.

**Figure 2:**
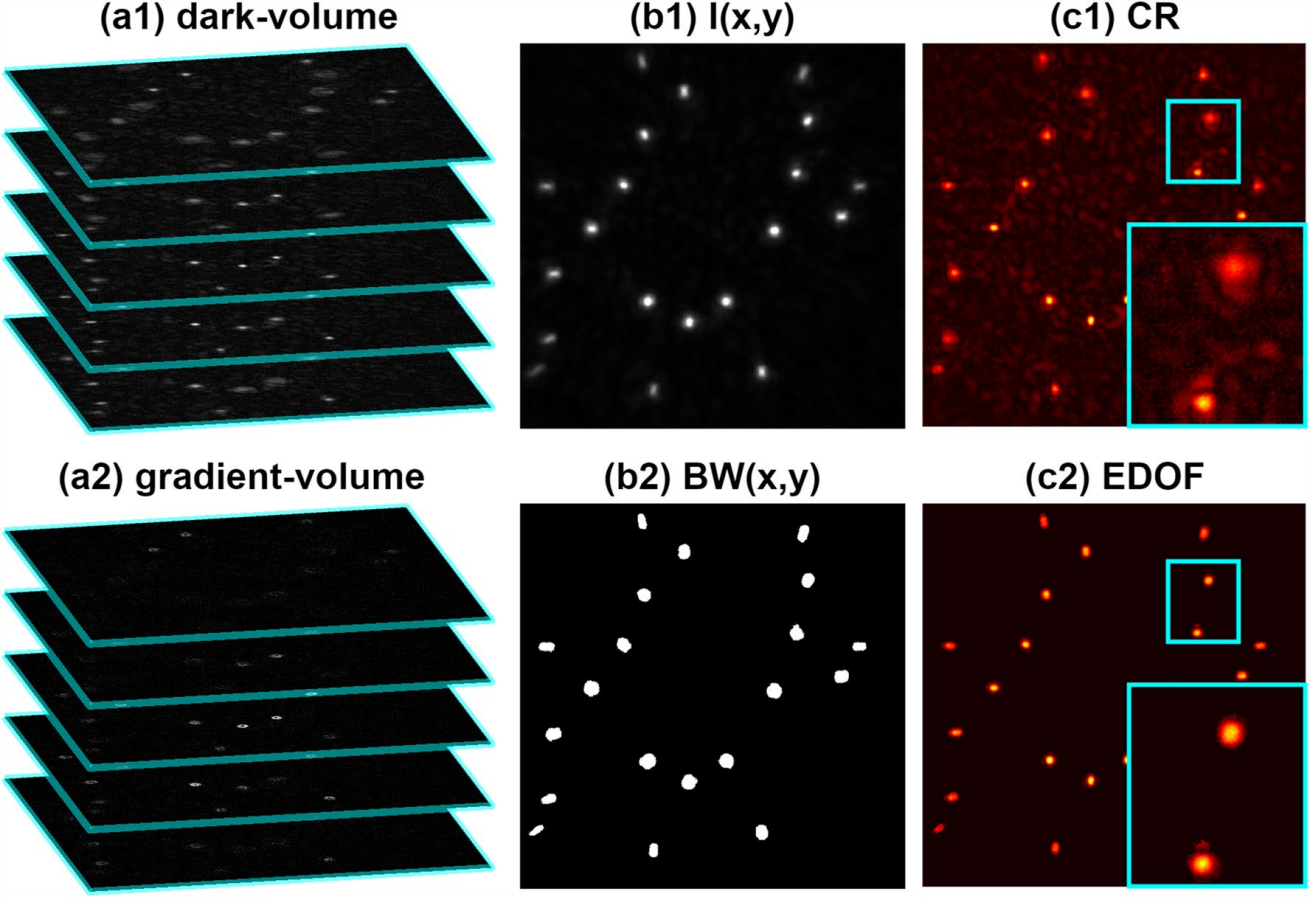
DarkTrack single-hologram (as shown in Fig. 1c2) processing scheme.

The novel DarkTrack algorithm, Fig. 2, is based on dark-volume DV(x, y, z)—3D high-contrast representation of the studied volume, computationally generated from a single Gabor hologram via its background term removal (see Supplement D) and AS numerical backpropagation to a predefined set of planes, Fig. 2a1 (Supplement C). Following the DarkFocus principle of operation^25^, we calculate the gradients of dark-volume in x/y directions and use them to create so-called gradient-volume GV(x,y,z)^2^, Fig. 2a2:

2 $$GV\left( {x,y,z} \right)^{2} = \left( {\frac{{\partial DV\left( {x,y,z} \right)}}{\partial x}} \right)^{2} + \left( {\frac{{\partial DV\left( {x,y,z} \right)}}{\partial y}} \right)^{2} .$$

The goal of the next step of DarkTrack is to mask all (x,y) areas that at some axial location z contain objects. To do that, we firstly compute global maxima maps of dark-volume and gradient-volume values along z axis (M_DV_(x, y) and M_GV_(x, y)) and use them to generate an image I(x, y), Fig. 2b1, to be binarized in a further step:

3 $$I\left( {x,y} \right) = M_{DV} \left( {x,y} \right) \times \left( {M_{GV} \left( {x,y} \right)} \right).$$

Via Eq. (3), we are combining information from M_DV_(x,y) and M_GV_(x,y). The M_DV_(x,y) has high values inside the regions occupied by objects, but also is sensitive to object-free regions disturbances (i.e., hologram noise and twin image effect), which makes it hard to binarize. M_GV_(x,y) enhances the visibility of the object borders while minimizing the values outside and inside the objects. Multiplying these two maps allows for enhancing the object information (and minimizing the signal in object-free regions) and, therefore, makes I(x,y) easy to binarize and get BW(x,y) via locally adaptive threshold value, accounting for varying object intensities, see Fig. 2b2.

Next, for each j-th object masked and segmented within the BW(x,y), we are calculating its (x_j_, y_j_, z_j_) locations. To set the depth coordinate $z_{j}$, we take the $M_{GV}$ values inside the j-th masked object (M_GVj_) and their corresponding $z$ locations Z_GVj_ and compute:

4 $$z_{j} = \frac{{\mathop \sum \nolimits_{x,y} M_{GVj} \left( {x,y} \right) \times Z_{GVj} \left( {x,y} \right)}}{{\mathop \sum \nolimits_{x,y} M_{GVj} \left( {x,y} \right)}}.$$

The (x_j_, y_j_) coordinates are calculated as the location of the pixel with maximum value of matrix DV(x,y,z_j_) inside the j-th object region masked by BW(x,y). We chose this method because of its simplicity and universality (ability to work independently of the object shape). However, when examining objects of known, well-defined (e.g., spherical) shapes, sophisticated sub-pixel algorithms may provide more accurate XY tracking^43^. When the coordinates of all objects are found, the EDOF image is computed by merging object dark field values taken from respective focal planes, Fig. 2c2. Compared to the classical reconstruction (CR—background-free hologram propagated to a selected plane with AS method), EDOF showcases all objects simultaneously in-focus and reduces twin-image artifacts. Elliptical shape of beads located near borders is governed by the space-domain-limited fringe content and does not significantly affect particle localization (see Supplement E for details).

**Figure 3:**
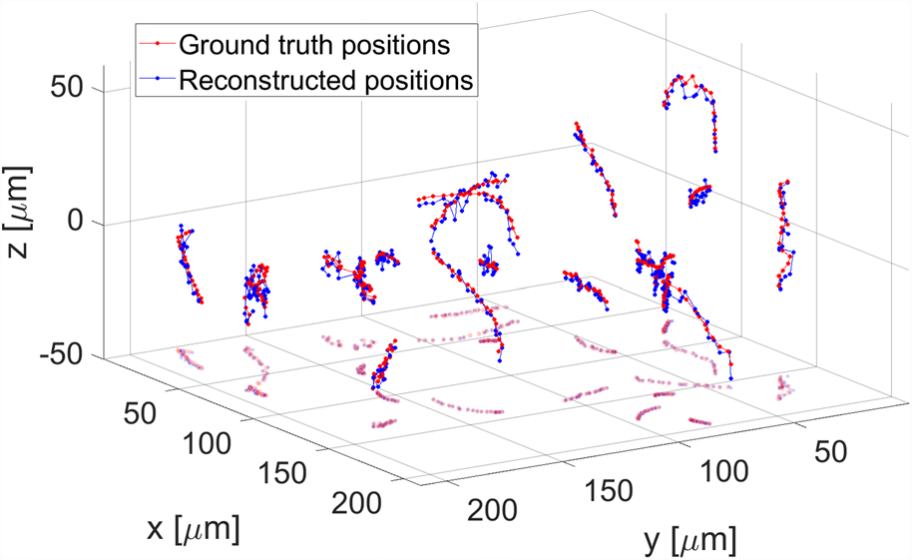
4D particle tracking reconstruction provided by DarkTrack for 20 holograms sequence (starting from Fig. 1c2).

The last step of DarkTrack algorithm consists in connecting the (x,y,z) positions for each segmented object in subsequent holograms (basing on the distances between the particles found in consecutive holograms and movement directions) to enable temporal tracking, Fig. 3. Visualization 1 and Visualization 2 show exemplary DarkTrack results obtained for simulated datasets^40^.

Proposed processing path provides many advantages such as short computation time (main load comes from hologram propagation to multiple planes; GPU acceleration further shortens the processing time to around 1 s for 500 × 500 × 121 DV analysis on computer with Intel Core i7-7700HQ 2.80 GHz processor, NVIDIA GeForce GTX 1060 graphics card and 32 GB RAM) and twin-image effect minimization. We have evaluated the tracking accuracy on several datasets provided by MSHoloSim and obtained RMS error of calculated positions smaller than half the diameter of microbeads for good hologram SNR and RMS smaller than a diameter for poor hologram SNR, see Table 1 for details. Those errors are smaller than, e.g., in Toloui & Hong^35^ (below 1.6 diameter) and in Pan & Meng^23^ (above half the diameter in Z direction for concentration of 6 particles/mm^3^ and above the particle diameter for concentration of 18 particles/mm^3^) methods. However, in its present form, DarkTrack is not suited for separating objects located at different depths which overlap in (x,y) plane. This feature, however, is common for all (also model-based) PT algorithms^16,20,35–37^.

**Table 1:** RMS error of microbead localization for varying SNR and number of microbeads (5 μm diameter; volume is set to yield high concentration of 2270 beads/mm^3^ and strong shadow density^44^ equal to 5675 for 10 beads case). RMS XY was calculated as: ${\text{RMS}}\left( {\sqrt {\left( {{\text{x }}{-}{\text{ x}}_{{{\text{ideal}}}} } \right)^{2} { } + { }\left( {{\text{y }}{-}{\text{ y}}_{{{\text{ideal}}}} } \right)^{2} } } \right)$.

SNR	10 beads		25 beads		50 beads		100 beads	
	RMS XY (μm)	RMS Z (μm)	RMS XY (μm)	RMS Z (μm)	RMS XY (μm)	RMS Z (μm)	RMS XY (μm)	RMS Z (μm)
Inf	1.5	1.58	1.64	2.47	1.66	3.16	1.93	3.45
20	1.53	1.69	1.65	2.80	1.67	3.34	2.03	3.63
10	1.55	2.25	1.69	3.03	1.71	3.65	2.10	3.80
5	1.59	2.71	1.63	3.63	1.69	3.6	2.10	3.69

## Experimental evaluation

**Figure 4:**
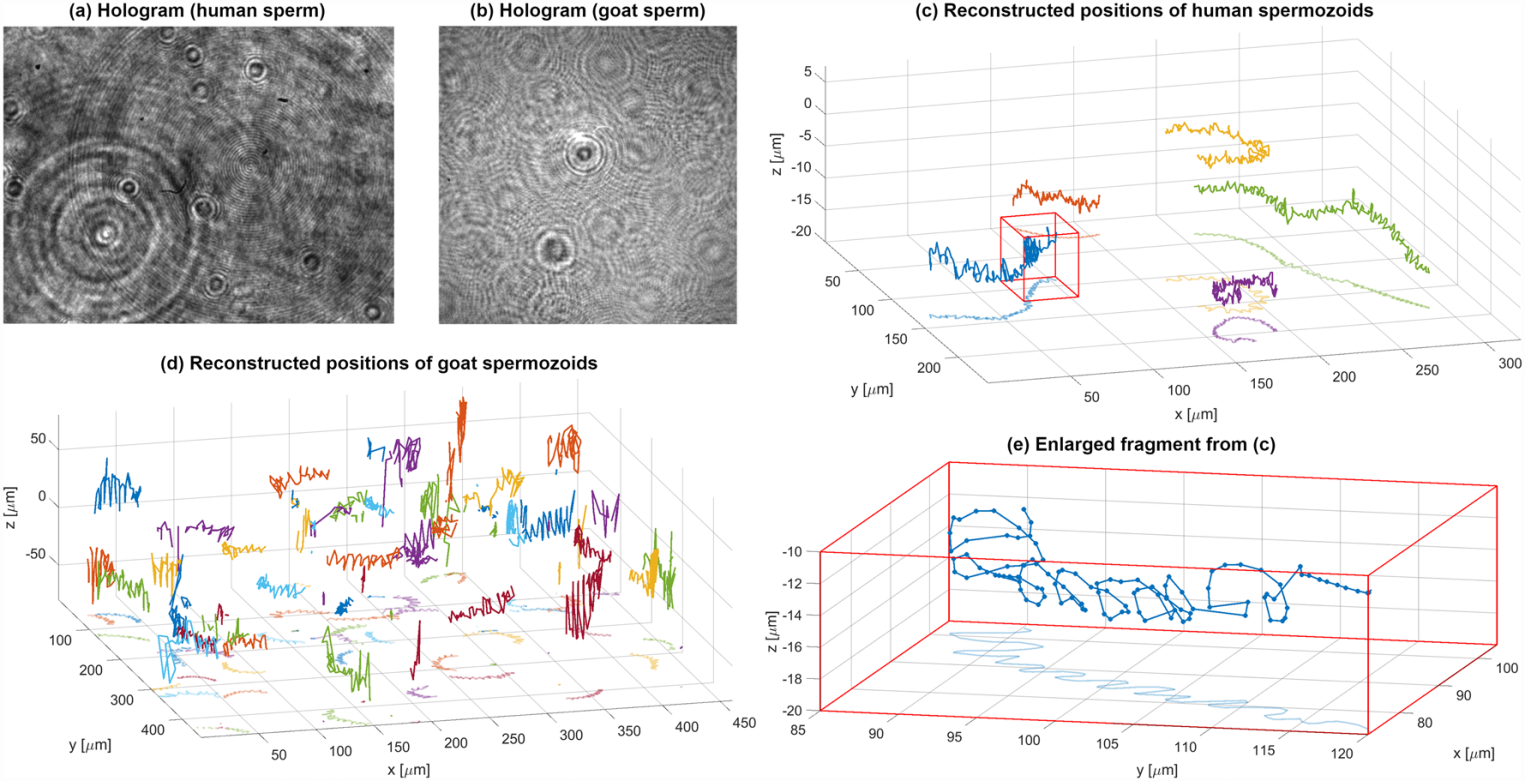
Live spermatozoid tracking results—human and goat specimen.

To experimentally validate our method (see Supplement F for holographic setups details), we examined human and goat live spermatozoa. We acquired two sequences of Gabor holograms utilizing in-line setups with objective lens (human sperm)^45^ and in lensless configuration (goat sperm)^46^, see exemplifying intensity distributions in Fig. 4a, b, respectively. For the lens-based case of human spermatozoids holograms were of high SNR and low number of slowly moving spermatozoids helped to treat this data as proof-of-concept analysis for very good imaging conditions. Results, presented in Fig. 4c, d, show characteristic spiral trajectories (commonly observed in literature^19^) accurately reconstructed via DarkTrack—see also Visualization 3 for dynamic tracking. Those processing results allow to establish the DarkTrack algorithm as recommendable for low-noise high-signal holograms captured for slowly moving sparse objects. Although it is a very interesting capability, there is a growing need to accurately examine the dynamics of large volumes densely filled by hastily moving objects. Goat spermatozoa, of much higher concentration and intense motility, constituted a challenging experimental case. Results presented in Fig. 4d and Visualization 4 demonstrate the DarkTrack’s ability to analyze dense volumes and track the 4D trajectories of multiple objects recorded via single holographic sequence. It is also to be note that, upon overlapping of multiple Gabor fringe patterns originating from large number of defocused spermatozoids, local signal to noise ratio has been decreased. Regardless of such diminished SNR, the DarkTrack was able to demultiplex information essential for robust tracking process.

## Discussion and conclusions

We now explain some implicit aspects of the proposed DarkTrack method. It is the first, to the best of our knowledge, openly-accessible model-free algorithm for precise 4D holographic in-line PT (space–time localization errors for challenging samples significantly below particle diameter). Applying DarkFocus metric, in terms of DV gradient, to each detected and segmented object yields more accurate results than other popular metrics such as in-focus criteria Dubois^26^ and Tamura^27^, as they in principle are very sensitive to region-of-interest selection and generally need bigger field of views (many more pixels) to act efficiently. Moreover, with DarkTrack both phase and amplitude objects can be assessed. The GPU acceleration provides fast calculations, dependent mainly on the number of planes constituting DV regardless object concentration. In addition, DarkTrack does not require any user-defined regularization parameters, therefore objects with different parameters (sizes, shapes, refractive indices, movement characteristics) could be tracked during single reconstruction. Moreover, the type of the sample is not needed to be known a priori, which highlights the versatility of the DarkTrack algorithm. The method for simulating holographic Gabor recording (MSHoloSim) accounts for multiple scattering, thus treating every synthetic object as a true 3D field and not as a 2D slice (or a stack of slices). Tracking 2D objects (in case of microbeads it would be 2D disks rather than 3D balls) is much less challenging and stays far from the experimental reality. The MSHoloSim generates reliable synthetic in-line holograms with known ground-truth particle coordinates for evaluation of 4D holographic PT. It is thus designed towards securing an important gap in holographic PT state-of-the-art and envisioned to serve as the first openly accessible tool for benchmarking and quantitative assessment of Gabor PT algorithms.

In summary, we derived a new holographic PT algorithm that uses a computationally rendered dark-volume and demonstrated it on datasets simulated via the novel numerical engine for closely mimicking in-line holographic microscopy (MSHoloSIM) as well as on experimental measurements. Our DarkTrack method, unlike its model-driven regularized counterparts, is prior-free, deterministic and does not require closely matching models and experimental conditions, thus is executed in much shorter time (from minutes in model-based algorithms^36^ to seconds, e.g., calculations for single hologram shown in Fig. 4b with 1088 × 1088 × 81 DV lasted 3,5 s) and provides versatile high tracking accuracy (with RMS error, in each direction, close to a half of the particle diameter). Moreover, it is applicable to lens-based and lensless DIHM and is not limited to microspheres as shown in experimental live spermatozoa tracking. New opportunities may arise from releasing *open access* codes for both DarkTrack algorithm and MSHoloSim engine^40^. We envision their positive impact on objective and reliable assessment of PT capabilities using Gabor in-line holography under different imaging conditions. We have also disseminated the data used throughout this work (dataset in^40^) to allow easy reproduction of the presented results and creditable comparison of existing and future in-line holographic PT approaches. It is interesting to note that DarkTrack working principle should also be applicable to dark-volumes coherently generated in off-axis holographic architecture and incoherently produced as a fluorescence microscopy z-stack.

## Supplementary Information

Supplementary Information 1.

Supplementary Video 1.

Supplementary Video 2.

Supplementary Video 3.

Supplementary Video 4.

## Data Availability

Data underlying the results presented in this paper are available in Ref.^40^.
