# Supplementary material for: Accurate automatic object 4D tracking in digital in-line holographic microscopy based on computationally rendered dark fields
Source: Sci Rep. 2022 Jul 28;12:12909. doi: 10.1038/s41598-022-17176-1 (PMC9334364; doi:10.1038/s41598-022-17176-1)
Supplement: Supplementary file 1 — Supplementary Information 1. [file 41598_2022_17176_MOESM1_ESM.pdf]

Accurate automatic object 4D tracking in digital in-line holographic microscopy based on computationally rendered dark fields: supplement

Mikołaj Rogalski,[1,3] Jose Angel Picazo-Bueno,[2] Julianna Winnik,[1] Piotr Zdańkowski,[1] Vicente Micó,[2] Maciej Trusiak[1,*]

[1]*Warsaw University of Technology, Institute of Micromechanics and Photonics, 8 Sw. A. Boboli St., 02-525 Warsaw, Poland*
[2]*Departamento de Óptica y de Optometría y Ciencias de la Visión, Universitat de Valencia, C/Doctor Moliner 50, Burjassot 46100, Spain*
[3]mikolaj.rogalski.dokt@pw.edu.pl
[*]maciej.trusiak@pw.edu.pl

A. Overview of available open-source in-line holographic particle tracking algorithms

Reconstructing the micro-object 4D (space + time) locations from lensless digital in-line holographic microscopy (DIHM) data is a task that was undertaken many times by numerous scientific groups. However, due to the lack of open-source software, researchers in this area are mainly focused on presenting a particular solution instead of comparing performance with other algorithms utilizing ground truth datasets. We would like to comment on 3 open-sources DIHM 4D tracking solutions published recently alongside with the codes. Unfortunately, due to reasons mentioned below, they are not fully suitable for comparing them with openly accessible DarkTrack.

The UmUTracker [1,2] – a graphical user interface application designed for 4D particle tracking from data acquired in light microscopy or DIHM. Thanks to the user-friendly interface, this program enables a straightforward reconstruction. However, because of the algorithmic method for finding the particle transverse (x,y) location that bases on the circle detection in hologram plane, it is not suitable for detecting objects inside the volume with high particle density (when the Gabor fringes from the neighboring particles are overlapping with each other). Moreover, for simpler holograms from our datasets (without Gabor fringes overlapping) the accuracy of the particle position at z direction exhibited errors greater than several particle diameters.

The MB-HoloNet [3] – a deep learning solution [4] for training neural networks for 3D volume reconstruction from a single hologram. Networks trained with MB-HoloNet architecture can reconstruct the full object volume within a short time (several milliseconds) and with very good quality. However, this solution is not universal (one neural network model is suitable only for a system/task with a certain, predefined parameters) and requires user to perform on its own extensive process of training neural networks. Moreover, MB-HoloNet is limited by the graphical processing unit (GPU) memory size, which in practice allows to train a model to reconstruct rather small volumes (training network to reconstruct 128x128x32 pixel volume requires over 15 GB GPU memory). Furthermore, MB-HoloNet provides a reconstructed 3D volume and requires users to further process it to retrieve objects 4D locations (outside the MB-HoloNet scheme).

The HoloFlow-PTV [5,6] – codes for reconstructing fluid flows from a set of DIHM holograms. In the HoloFlow-PTV, fluid flows are reconstructed basing on the reconstructed particle volume and strong priors on how these particles are allowed to move (e.g., divergence-free flow). In practice, it makes the algorithm truly valuable and capable for studying

fluid flows (as it was carefully designed for), however, applying it to data where the particles are allowed to rapidly change movement directions (as in our case of somewhat randomized trajectories) may cause reconstruction errors.

B. Beam propagation method

Beam propagation method (BPM) is a class of algorithms that allows for optical field propagation in inhomogeneous refractive index distribution. In our work, we have used the BPM for propagation of 2D field $u(x, y, 0)$ along z-axis through a discretized 3D object volume following the Algorithm 1 [7]:

Algorithm 1: BPM for propagating the optical field through object volume

for z_i from 0 to S_z with axial step δz:

$$u(x, y, z_i + \delta z) = e^{\frac{j2\pi(n(x,y,z_i+\delta z)-n_0)\delta z}{\lambda}} \times F^{-1}\left(F[(u(x, y, z_i)] \times e^{-j2\pi\delta z\left(\frac{f_x^2+f_y^2}{\frac{n_0}{\lambda}+\sqrt{\left(\frac{n_0}{\lambda}\right)^2-f_x^2-f_y^2}}\right)} \right),$$

where S_z is the volume size in z direction, n_0 is the background refractive index, i.e., refractive index of the surrounding medium, λ is the light wavelength, f_x and f_y are the spatial frequencies along x and y axes, $n(x, y, z)$ is the object complex refractive index distribution, which real part is responsible for phase delay and imaginary part (extinction coefficient) defines the light absorption; F and F^{-1} stands for 2D Fourier transform and inverse Fourier transform, respectively.

C. Angular spectrum method

Angular spectrum method (AS) is an algorithm that propagates the optical field $(u(x, y, z))$ in the free-space at Δz distance according to Equation (S1) [8,9]:

$$u(x, y, z + \Delta z) = F^{-1}\left(F\big(u(x, y, z)\big) \times e^{j2\pi\Delta z\sqrt{\left(\frac{n_0}{\lambda}\right)^2-f_x(x,y)^2-f_y(x,y)^2}} \right). \tag{S1}$$

D. Strategies for removing holograms background component

Reconstructing a background free hologram (i.e., hologram with diffraction fringes oscillating around 0) is a common technique in DIHM as it allows for high contrast object reconstruction with mixed amplitude and phase factors. Computing the hologram background may be performed in several ways, e.g., by setting image collected without object presence in the system as the background [10], or by high-pass filtering each hologram with either the simple Gaussian kernel [11] or more sophisticated fringe pattern filtering methods like the empirical mode decomposition algorithm [12,13] or the recently proposed iterative filtering algorithm [14,15]. In the DarkTrack algorithm, we set as the background the mean frame from all collected holograms due to this solution simplicity and robustness. Moreover, it enables removing static objects in the processing path, which improves the results readability. In the case of processing datasets consisting of a low number (below 10) of holograms or when there is a need to observe both static and dynamic objects, we extract the background by filtering holograms with the classical Gaussian filter with sigma parameter equal to 30, that enables satisfactory background filtration for high variety of the holograms. Efficiency of such simple filtering further corroborates robustness of the proposed DarkTrack algorithm.

E. Interpretation of the near edge microbeads elliptical shape occurrence

When recording a lensless microscopy hologram, the information about the object is stored in the Gabor fringes, which result from the interference between light that seamlessly passes through the sample and light that is scattered by the sample, Fig. S1(a). Back propagating this hologram to the object plane allows for recovering the object information, Fig.

S1(b) and the more Gabor fringes are present in the hologram, Fig. S1(c), the more object information may be reconstructed and, therefore, the higher resolution reconstruction is obtained, Fig S1(d).

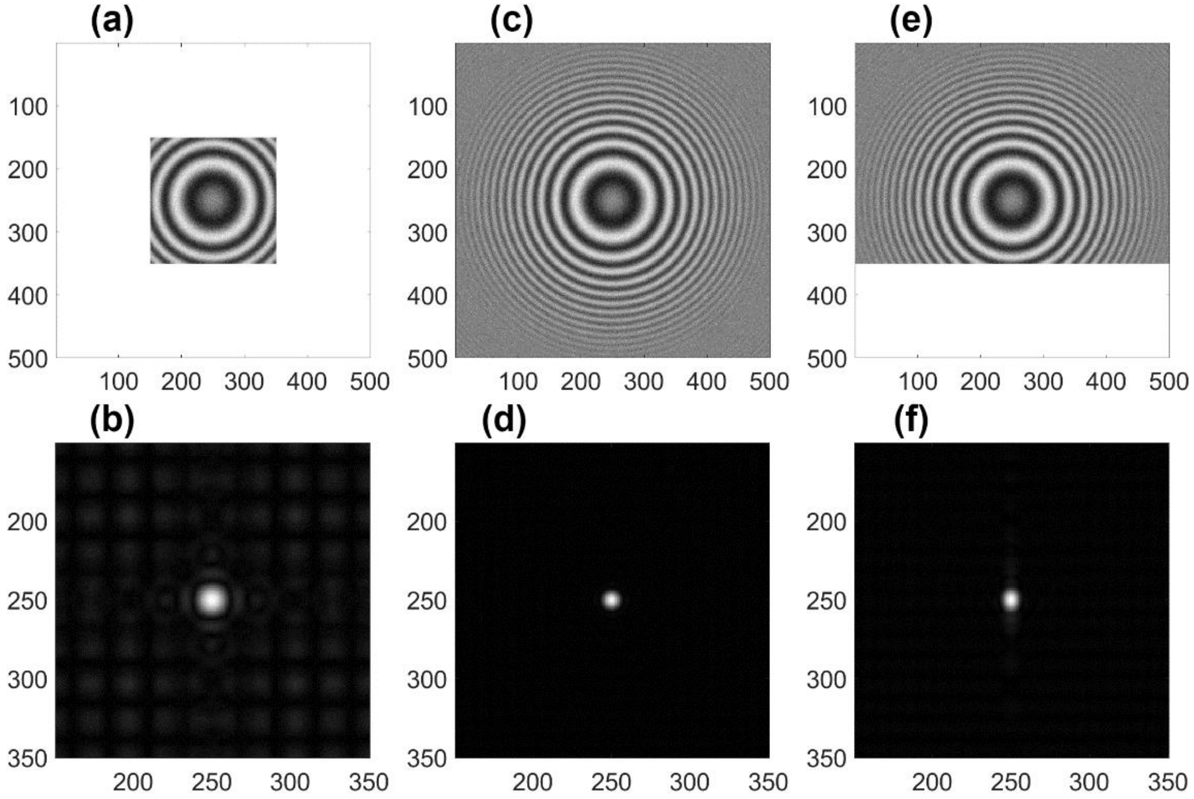

Figure S1. Simulation results. Simulated holograms (a),(c),(e) and their reconstructed dark field amplitudes (b),(d),(f) respectively.

In the case of observing an object that is close to the image edge, its hologram Gabor fringes are cut by the camera aperture from a given side, Fig. S1(e). This results in the reconstruction resolution drop along the direction perpendicular to this edge, which causes the reconstruction directional blur, and therefore an elliptical shape of the reconstructed spherical microbead, Fig. S1(f). However, this blur is symmetrical with respect to the microbead center, hence, this effect does not affect the accuracy of finding microbead (x,y) center employed in the proposed DarkTrack algorithm.

F. Description of the experimental systems

For the experimental validation of our DarkTrack method involving live spermatozoa, we implemented two different optical systems. On one hand, a lens-based DIHM scheme, Fig. S2(a), is defined for human sperm analysis [16]. Essentially, it includes the embodiment of an upright bright-field microscope (Olympus BX60), having an UMPlanFl 20X/0.46NA microscope objective and a tube lens system as imaging optics. For DIHM implementation, we externally inserted a coherent blue (450 nm) illumination coming from a fiber coupled laser diode from Blu Sky Research (SpectraTec 4 STEC4 405/450/532/635 nm), and the sample is axially shifted 90 μm from its conventional imaging position (see [16] for more details). Finally, the in-line holograms are recorded using a digital camera (Mightex USB 3.0 SMN-B050-U, CMOS sensor type, 2560x1920 pixels, 2.2x2.2 μm pixel pitch).

On the other hand, goat spermatozoa are studied with a lensless DIHM [17]. Here, a violet (405 nm) light coming from a Blu-ray optical unit, is focused using a high NA lens to provide a coherent quasi-point source. Then, such a light passes through the sample and the magnified (M = 15 X) Gabor hologram is recorded with the same digital camera (Mightex CMOS sensor), Fig. S2(b).

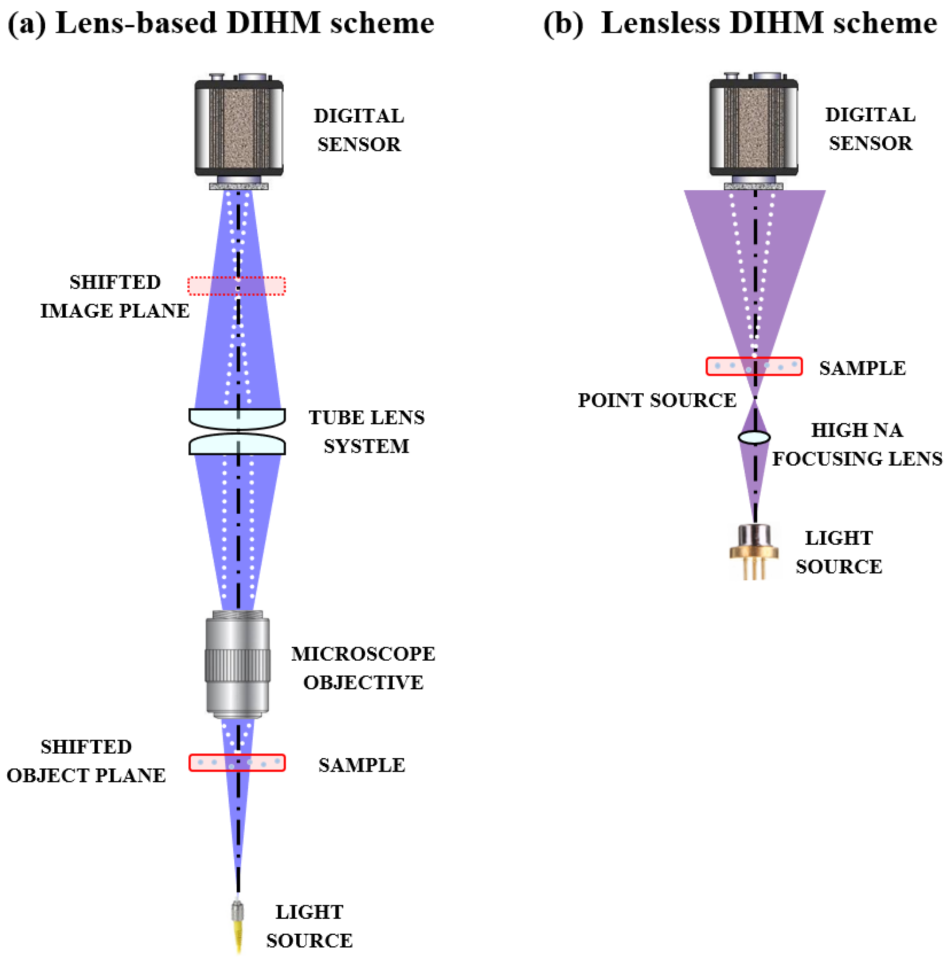

Figure S2. Optical schemes of the (a) lens-based and (b) lensless DIHM setups implemented for DarkTrack validation involving human and goat spermatozoa, respectively.

References

1. H. Zhang, T. Stangner, K. Wiklund, A. Rodriguez, and M. Andersson, "UmUTracker: A versatile MATLAB program for automated particle tracking of 2D light microscopy or 3D digital holography data," Comput. Phys. Commun. **219**, 390–399 (2017).
2. https://sourceforge.net/projects/umutracker
3. https://github.com/ni-chen/3D-MB-HoloNet.
4. Y. Wu, Y. Rivenson, Y. Zhang, Z. Wei, H. Günaydin, X. Lin, and A. Ozcan, "Extended depth-of-field in holographic imaging using deep-learning-based autofocusing and phase recovery," Optica **5**, 704-710 (2018).
5. N. Chen, C. Wang, and W. Heidrich, "Snapshot Space–Time Holographic 3D Particle Tracking Velocimetry," Laser Photonics Rev. **15**, 2100008 (2021).
6. https://github.com/Ni-Chen/HoloFlow-PTV
7. U. S. Kamilov, I. N. Papadopoulos, M. H. Shoreh, A. Goy, C. Vonesch, M. Unser, and D. Psaltis, "Optical Tomographic Image Reconstruction Based on Beam Propagation and Sparse Regularization," IEEE Trans. Comput. **2**, 59-70 (2016).
8. M. K. Kim, "Principles and techniques of digital holographic microscopy," J. Photonics Energy **1**, 018005 (2010).
9. T. Latychevskaia and H.-W. Fink, "Practical algorithms for simulation and reconstruction of digital in-line holograms," Appl. Opt. **54**, 2424 (2015).
10. H. Tobon, C. Trujillo, and J. Garcia-Sucerquia, "Preprocessing in digital lensless holographic microscopy for intensity reconstructions with enhanced contrast," Appl. Opt. **60**, A215 (2021).
11. A. Dogra and P. Bhalla, "Image Sharpening By Gaussian And Butterworth High Pass Filter," Biomed. Pharmacol. J. **7**, 707–713 (2014).
12. M. Trusiak, J.-A. Picazo-Bueno, P. Zdankowski, and V. Micó, „DarkFocus: numerical autofocusing in digital in-line holographic microscopy using variance of computational dark field gradient," Opt. Lasers Eng. **134**, 106195 (2020).

13. M. Trusiak, M. Wielgus, and K. Patorski, "Advanced processing of optical fringe patterns by automated selective reconstruction and enhanced fast empirical mode decomposition," Opt. Lasers Eng. **52**, 230–240 (2014).
14. A. Cicone and H. Zhou, "Multidimensional Iterative Filtering Method for the Decomposition of High–Dimensional Non–Stationary Signals," Numer. Math. Theory, Methods Appl. **10**, 278–298 (2017).
15. M. Rogalski et al., "Tailoring 2D fast iterative filtering algorithm for low-contrast optical fringe pattern preprocessing," Opt. Lasers Eng. **155**, 107069 (2022).
16. V. Micó, K. Trindade, and J. Á. Picazo-Bueno, "Phase imaging microscopy under the Gabor regime in a minimally modified regular bright-field microscope," Opt. Express **29**, 42738-42750 (2021).
17. J. A. Picazo-Bueno, K. Trindade, M. Sanz, V. Micó, "Design, Calibration, and Application of a Robust, Cost-Effective, and High-Resolution Lensless Holographic Microscope," Sensors **22**, 553 (2022).
